# The optimization of crop response to climatic stress through modulation of plant stress response mechanisms. Opportunities for biostimulants and plant hormones to meet climate challenges.

**DOI:** 10.1111/nph.70701

**Published:** 2025-11-10

**Authors:** Jing Li, Giulia Forghieri, Danny Geelen, Patrick du Jardin, Patrick H. Brown

**Affiliations:** ^1^ HortiCell, Department Plants and Crops, Faculty of Bioscience Engineering Ghent University Coupure Links 653 Ghent 9000 Belgium; ^2^ CatMat Lab, Department of Molecular Sciences and Nanosystems Ca’ Foscari University of Venice and Consortium INSTM UdR VE via Torino 155 Venice 30172 Italy; ^3^ Plant Biology Laboratory, Gembloux Agro‐Bio Tech University of Liège Passage des Déportés 2 Gembloux 5030 Belgium; ^4^ Department of Plant Sciences University of California Davis One Shields Ave Davis 95616 CA USA

**Keywords:** abiotic stress, biostimulant, climate change, plant hormone, stress tolerance

## Abstract

This review discusses the use of agronomic management practices to enhance crop stress resilience to climate stress through the modulation of natural plant growth regulatory pathways. The use of biostimulants or plant hormones to improve crop resilience is subject to strict regulatory oversight if changes in the regulation of plant growth are implied. Climate change is a major threat to crop potential and is characterized by both long‐term shifts in temperature and precipitation patterns as well as increased occurrence of extreme weather events, posing an immediate threat to agriculture. Breeding and exogenous inputs have been used to enhance cropping system resilience, although these management practices are either too slow or constrained by cost and availability, to address rapidly emerging climate challenges. Exogenous biostimulants, microbials and plant hormones have shown great promise as novel mechanisms to optimize natural plant resilience, resulting in immediate but non‐permanent improvements in plant responses to climate‐induced stresses, representing a powerful but underexplored approach to enhance crop productivity under climate stress. The use of these exogenous inputs is, however, constrained by outdated and scientifically unsound regulations that consider any such modification as pesticidal in nature. The failure to modernize regulatory frameworks for the use of biostimulants in agriculture will constrain the development of safe effective tools and deprive growers of means to respond to climate change. Here, we discuss the scientific rationale for eliminating the regulatory barriers governing biostimulants or products that modulate plant regulatory networks and propose a framework for enabling legislation to strengthen cropping system resilience.

## Introduction

The United Nations issued a red alert in 2023 after new records were set for every major climate indicator (WMO, 2024). Recent projections indicate that the effects of climate change will emerge earlier than expected, with several major crop‐producing regions likely to experience significant impacts before 2040 (Jägermeyr *et al*., 2021). By 2050, it is projected that an additional 20% of the global population could face hunger due to the impact of a once‐in‐100‐yr extreme climate event (Hasegawa *et al*., 2021). As erratic and extreme weather patterns intensify, traditional farming systems are becoming increasingly vulnerable.

Changes in average temperature and precipitation affect crop adaptability by influencing photosynthesis, respiration and water use, while the increased frequency and intensity of climate extremes pose more complex physiological and agronomic challenges. In Brazil, for example, weather variability over the past two decades has caused a 50% increase in yield fluctuations for major crops (Burney *et al*., 2024). Extreme short‐term events – such as frost, heat, drought and flooding – pose a particular disruption to cropping systems. Examples include wet or cold springs, which delay cereal crop planting, reduce germination and emergence and shorten the growing season, ultimately lowering yields. Insufficient winter chill disrupts flowering in temperate trees, reducing fruit set. Heat spells during flowering impair seed set in many crops. Erratic rainfall and unusual heat patterns extend and weaken flowering in tropical species like coffee, resulting in uneven ripening, increased harvesting costs and reduced yield and quality. These extreme events not only affect crop physiology but also disrupt routine farming practices, adding costs, risks and reduced profitability to growers.

To cope with climate stress, native species have evolved highly sophisticated adaptive plasticity that enables them to respond effectively to environmental changes (Brooker *et al*., 2022). Plant adaptive plasticity refers to the physiological mechanisms that allow plants to adjust to growth‐limiting resource shortages in variable environments. Many modern crop species, however, are significantly less tolerant of climate variability compared to their wild relatives (Quezada‐Martinez *et al*., 2021; Fumia *et al*., 2022; Toulotte *et al*., 2022; Landis *et al*., 2024). The focus on selecting a limited number of high‐yielding, commercially valuable cultivars has led to crop genetic erosion, diminishing both the adaptive plasticity and genetic diversity necessary to cope with climate change (Khoury *et al*., 2022). Excessive use of certain agricultural chemicals and the consequent degradation of soil health have also adversely affected beneficial plant‐associated microbiomes and further deteriorated soil quality, thereby undermining the resilience of cropping systems (Liu *et al*., 2025).

Adaptive plasticity is in part mediated through plant hormones. Plant hormones are signaling molecules that regulate physiological processes and developmental programs in response to both endogenous signals and environmental cues (Lichtfouse, 2021). Extensive crosstalk among plant hormones establishes a complex regulatory network that fine‐tunes the balance between growth and stress responses. The centrality of plant hormone pathways in crop stress resilience suggests that the targeted modification of plant hormone response networks will be a critical strategy in the development of more resilient crops. Modification of plant hormone response networks to improve crop stress response has historically been achieved through breeding and through increased agronomic inputs (irrigation, fertilization, crop protection, etc.) and more recently through the application of plant biostimulants, plant hormones and microbial products (Brown & Saa, 2015; Eshed & Lippman, 2019; Rouphael & Colla, 2020; Hirayama & Mochida, 2022). Each approach has distinct benefits and constraints.

Crop breeding is a strategy that has long been employed to improve abiotic stress tolerance, though with mixed success due to its complexity (Cagnola *et al*., 2025). Climate change impacts are variable, multifactor, often localized and cropping system‐specific and as a result, they are slow and challenging to address with breeding strategies. Extreme weather events induced by climate change are unpredictable and often highly localized, and as such, they are extremely difficult to select or breed for. Breeding for stress tolerance is further hindered by our limited understanding of the genetic mechanisms underlying these traits, while the uncertainty of climate change‐induced disruptions makes trait selection difficult.

Climate stress tolerance can also be managed through increased crop inputs (water, nutrients and soil amendments) and through improved management technologies, such as conservation tillage, shading and frost prevention, each of which can help mitigate environmental stress. While these approaches can be effective, rapid and flexible, they are time‐intensive and depend upon the availability and cost‐effectiveness of the needed inputs.

The application of exogenous chemicals including microbial products (Zhang *et al*., 2021), biofertilizers and biostimulants for the management of plant stress responses is an area of tremendous interest and unmet potential that has the added benefit of being rapid and targeted with a generally low cost of implementation. The use of biostimulants and plant hormones to achieve climate stress tolerance is however, strongly constrained by a lack of understanding of the mechanisms involved (Walia, 2023) and by regulatory restrictions that constrain the use of any product that explicitly targets plant growth and development processes. This constraint applies even if the changes in plant growth and development or plant hormone levels that result from product application do not differ from those that occur naturally in well‐adapted species.

While biostimulants have been gaining acceptance as a mechanism to enhance crop resilience, many biostimulant products have been observed to have variable benefits, resulting in commercial uncertainty which constrains adoption (du Jardin *et al*., 2025). This occurs in part because biostimulant response depends on unpredictable plant stress events and complex soil and genetic interactions. Uncertainty is exacerbated by inadequate understanding of modes of action, in part because regulatory constraints discourage producers from pursuing a full understanding of the mechanisms involved for fear of disclosing plant growth regulatory effects.

This review examines the mechanism of plant response to the environment and tolerance to climate stress, contrasts three strategies to address climate stress and highlights both the similarities, opportunities and constraints of these approaches. The implication of these approaches for the development of sound regulatory frameworks governing the use of plant hormones and biostimulants in sustainable agriculture is discussed.

## The mechanisms of plant stress response

**Fig. 1:**
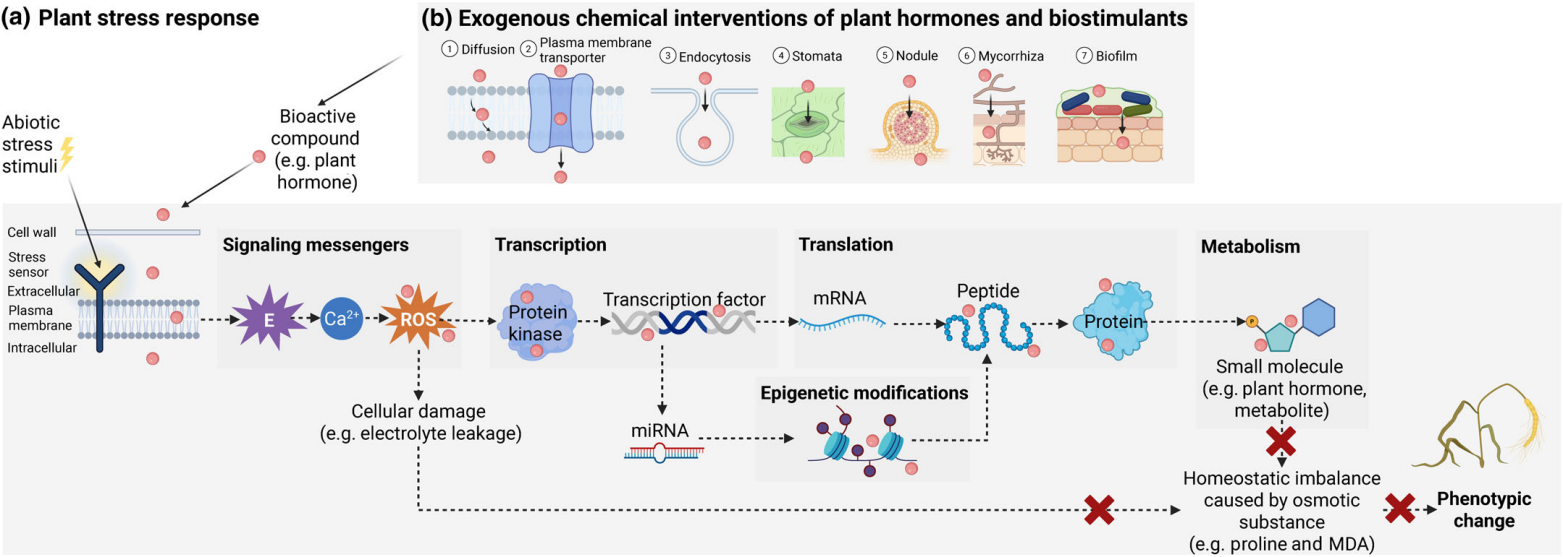
The mechanisms underlying plant stress responses (a) and the roles of exogenous bioactive compounds in modulating cascade pathways (b). Plant hormones and biostimulants are examples of bioactive compounds that can be either derived from natural substances or chemically synthesized (Ca^2+^, calcium ions; E, electrical signal; ROS, reactive oxygen species; mRNA, messenger RNA; MDA, malondialdehyde). Solid arrows indicate the exogenous processes involved when applying bioactive compounds under plant stress conditions. Dashed arrows indicate the corresponding endogenous plant responses to the application. The X mark indicates the possible functions of exogenous bioactive compounds in abiotic stress mitigation.

Plants respond to abiotic stress through a complex cascade of signaling events, starting with perception and signal transduction, followed by the induction of stress‐related genes and downstream processes (Fig. 1a). Early environmental signals are sensed by plants or their microbial root‐associated partners, which then are converted into chemical messages, including biosynthetic compounds such as phytohormones and other bioactive metabolic byproducts, that transduce from the cellular level to the organ and ultimately to the whole plant (Zhang *et al*., 2021). Upon stress perception, rapid changes occur in plant response pathways and their regulatory networks, including second messengers, transcriptional reprogramming, transcript processing and post‐translational protein modifications. This regulatory signaling network also governs primary and secondary metabolism, such as plant hormones (Sulpice & McKeown, 2015). Our understanding of plant stress response pathways is complicated by the nonlinear nature of responses across environmental gradients and phenological stages (Arnold *et al*., 2019). Moreover, epigenetic mechanisms that enable plants to adapt through priming and stress‐dependent memory add complexity, creating a temporal disconnect between the observed stress response and the current stress condition (Gallusci *et al*., 2023).

Plant cells and their subcellular compartments have specialized sensors or sensory systems that detect early deviations from homeostasis in response to stress (Dietz & Vogelsang, 2024). During signal transduction, spikes and waves of electrical signals and secondary messengers, such as Ca^2+^ and reactive oxygen species (ROS, such as H_2_O_2_), function as signaling molecules under normal conditions. Proline accumulates as an osmotic substance during drought stress, helping sustain photosynthetic electron transport (Alvarez *et al*., 2022). Similarly, free malondialdehyde (MDA) levels rise under stress conditions during the lipid peroxidation process, induced by ROS and as a consequence of increased lipoxygenase activity (Morales & Munné‐Bosch, 2019). Non‐enzymatic antioxidants scavenge free radicals or indirectly regulate core metabolic enzymes to mitigate oxidative damage (Mittler *et al*., 2022). Downstream protein kinases, regulated via central metabolism, coordinate the growth–defense tradeoff by managing resource allocation (He *et al*., 2022). These include well‐characterized pathways such as the Sucrose non‐fermentable 1‐related protein kinase 1 (SnRK1) and the target of rapamycin (TOR) pathways (Baena‐González & Hanson, 2017). Stress‐related gene expression can also be regulated by transcription factors, with activity strongly dependent on stress intensity. Elevated transcript levels of these genes can disrupt normal RNA processing, which in turn modulates translation and post‐translational modifications, ultimately leading to altered localization and production of peptides, proteins and small molecules (Zhang *et al*., 2021). Additionally, epigenetic modifications, including DNA methylation, chromatin remodeling, synthesis of regulatory small RNA molecules and histone dynamics, may also occur (Chang *et al*., 2020). Central metabolism mediates endogenous hormone biosynthesis and regulates other functions – such as metabolism, perception, signaling and transport – at the intersection of plant stress responses (Fàbregas & Fernie, 2021).

## Plant hormones in plant stress response

**Fig. 2:**
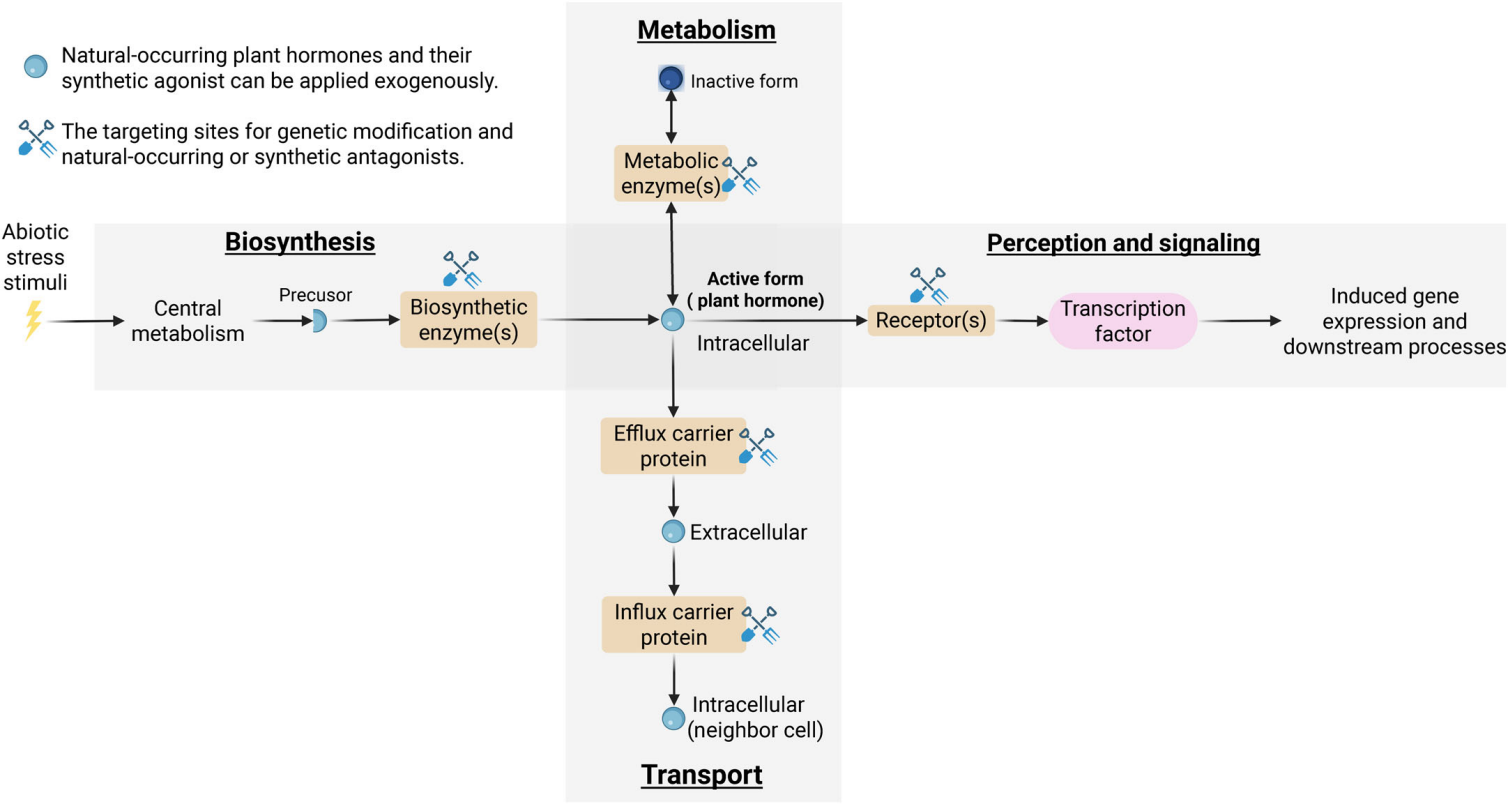
Hormone homeostasis is mediated through biosynthesis, metabolism, transport, signaling and perception.

The central role of plant hormones in stress perception, signaling and responses suggests that there is potential to target the modification of hormone signaling pathways for improved resilience. In plants, cellular activity is fine‐tuned through local adjustments in hormone levels, which are dynamically regulated by biosynthesis, catabolism, transport and signal perception (Fig. 2). The active forms of plant hormones are synthesized from precursor molecules derived from primary metabolites, such as amino acids and nucleotides, or converted from more complex secondary metabolites through specific enzymes that are responsible for biosynthetic and catabolic steps. Hormone homeostasis is further regulated by conjugation with sugars or amino acids, often, but not always, resulting in inactive hormone derivatives.

Plant cells express various hormone receptors that trigger physiological responses, such as proton pump activation, or initiate downstream signaling to regulate gene transcription. Natural plant hormones are highly mobile, moving between cells through the apoplast via efflux and influx carrier proteins or through plasmodesmata connecting adjacent cells. This mobility allows hormones to be transported between tissues and organs, such as from roots to shoots. Additionally, hormones can travel long distances through vascular tissues, influencing their spatial and temporal activity. Each aspect of hormone transport and signaling presents opportunities for targeted modification using genetic tools or exogenous bioactive compounds to fine‐tune plant responses.

### Genetic approaches to enhance plant stress tolerance through hormone pathway modifications

Over the past decades, extensive efforts have been made to engineer hormone signaling pathways through breeding approaches, yet examples of commercial implementation are limited (Table 1). A diverse array of mutants modulating plant hormone pathways has been identified, targeting biosynthetic and metabolic enzymes, receptors and transporters (Fig. 2). Table 1 summarizes loss‐of‐function (reduced or abolished protein activity) and gain‐of‐function mutations (enhanced protein activity) that alter hormone pathways and influence crop phenotypic changes under stress conditions. The effects of these mutations on stress tolerance vary on a case‐by‐case basis. For example, *OsGH3‐2* overexpression in rice, which encodes an enzyme catalyzing IAA (a natural auxin) conjugation, enhances cold tolerance but increases drought sensitivity due to its modulation of both IAA and abscisic acid homeostasis (Du *et al*., 2012). Similarly, cytokinin‐deficient potato lines overexpressing *AtCKX2* (Arabidopsis thaliana Cytokinin Oxidase/Dehydrogenase2) exhibit improved tolerance to mild salinity stress but reduced tolerance to severe salinity stress compared to the wild‐type (Raspor *et al*., 2024).

**Table 1:** Molecular manipulation of plant hormone pathways and their influence on crop stress responses.

Plant hormone	Mutant variation	Treated crop	Stress condition	Mechanism involved	Genetic technique and target gene	Phenotype changes
Auxin	Loss‐of‐function	Tomato	Drought	Signaling	Deficient *ARF4* gene encoding Auxin‐signaling F‐Box protein	Vegetative growth↑ (Chen *et al*., 2021)
			Salt			Vegetative growth↑ (Bouzroud *et al*., 2020)
		Tomato	Cadmium		Deficient auxin‐insensitive DIAGEOTROPICA (DGT) gene	Vegetative growth↓ (Alves *et al*., 2017)
		Rice	Heat		Deficient *IAA29* gene encoding AUX/IAA protein	Grain yield↓ (Chen *et al*., 2024)
		Rice	Aluminum	Transport	Deficient *AUX3* gene encoding AUXIN1/LIKE AUX1 protein	Seedling growth↑ (M. Wang *et al*., 2019)
		Rice	Cadmium		Deficient *AUX1* gene encoding AUXIN1/LIKE AUX1 protein	Seedling growth↓ (Yu *et al*., 2015)
		Rice	Cold and drought		Deficient *PIN1b* gene encoding PIN protein	Vegetative growth↓ (Yang *et al*., 2023)
	Gain‐of‐function	Rice	Drought	Biosynthesis	Overexpressing *GH3* gene encoding IAA‐amido synthetase	Seedling growth↓ (Du *et al*., 2012)
			Cold			Seedling growth↑ (Du *et al*., 2012)
Abs*cis*ic acid	Loss‐of‐function	Tomato	Drought	Biosynthesis	Deficient *notabilis/flacca (not/flc)* double mutant that frameshift mutation in the NCED1 gene	Fruit size↓ (Nitsch *et al*., 2012)
		Barley	Drought		Deficient Az34 mutant (*nar2a* gene) encoding molybdopterin	Seedling growth↓ (Walker‐Simmons *et al*., 1989)
		Tomato	Drought		Deficient *Sitiens* gene encoding dehydrogenase	Vegetative growth↓ (Harrison *et al*., 2011)
		Tomato	Drought or salt		Deficient *flacca* gene encoding dehydrogenase	Vegetative growth↓ (Grillo *et al*., 1995)
		Potato	Drought		Sensitive mutant blocked at ABA‐aldehyde	Vegetative growth↓ (Etehadnia *et al*., 2008)
		Tobacco	Drought		Deficient *aba1* gene encoding zeaxanthin epoxidase	Vegetative growth↑ (Mizokami *et al*., 2015)
	Gain‐of‐function	Tomato	Salt	Biosynthesis	Overexpressing the NCED1 gene encoding enzyme 9‐*cis*‐epoxycarotenoid dioxygenase	Vegetative growth↑, fruit yield↑ (Martínez‐Andújar *et al*., 2021)
		Tobacco	Drought			Vegetative growth↑ (Qin & Zeevaart, 2002)
Brassinosteroid	Loss‐of‐function	Tomato	Heat	Signaling	Insensitive curl3^−abs^ gene	Seedling growth↓ (Mazorra *et al*., 2011)
	Gain‐of‐function	Tomato	Heat	Biosynthesis	Overexpressing *Dwarf* gene	Seedling growth↑ (Mazorra *et al*., 2011)
		Oilseed	Drought or heat		Overexpressing *DWF4* gene encoding C‐22 hydroxylase	Vegetative growth↑, grain yield↑ (Sahni *et al*., 2016)
		Tomato	Cold	Signaling	Overexpressing BRI1 gene encoding BR receptor kinase	Vegetative growth↑ (D. Wang *et al*., 2022)
		Maize	Salt		Overexpressing *BSK1* gene encoding BR‐signaling kinase	Vegetative growth↑ (Liu *et al*., 2022)
		Creeping bentgrass	Drought	Metabolism	Overexpressing *BAT1* gene encoding BR‐related acyltransferase	Vegetative growth↑ (Han *et al*., 2017; Han *et al*., 2017)
Cytokine	Loss‐of‐function	Tomato	Drought or heat	Signaling	Downregulating *HK2* gene encoding histidine kinase	Vegetative growth↑ (Mushtaq *et al*., 2022)
		Tomato	Salt		Downregulating *AHP* gene encoding histidine phosphotransfer protein	Seedling growth↓ (Sun *et al*., 2014)
	Gain‐of‐function	Creeping bentgrass	Heat	Biosynthesis	Overexpressing *ipt* gene encoding isopentenyltransferase	Vegetative growth↑ (Xu *et al*., 2009)
			Drought			Vegetative growth↑ (Xu *et al*., 2016)
		Barley	Drought	Metabolism	Overexpressing *CKX1* gene encoding cytokinin dehydrogenase	Vegetative growth↑ (Pospíšilová *et al*., 2016)
		Tobacco	Drought			Vegetative growth↑ (Lubovská *et al*., 2014)
		Potato	Salt (mild)		Overexpressing *CKX2* gene encoding cytokinin dehydrogenase 1	Seedling growth↑ (Raspor *et al*., 2024)
			Salt (severe)			Seedling growth↓ (Raspor *et al*., 2024)
		Rice	Drought or salt		Overexpressing *LOG* gene encoding phosphoribohydrolase	Vegetative growth↑, grain yield↑ (Rathore *et al*., 2024)
Ethylene	Loss‐of‐function	Tobacco	Salt	Biosynthesis	Deficient ACS gene encoding 1‐aminocyclopropane‐1‐carboxylate synthase	Vegetative growth↑ (Wi *et al*., 2010)
	Gain‐of‐function	Rice	Drought and	Biosynthesis	Overexpressing ET overproducer gene ETOL1	Vegetative growth↑ (Du *et al*., 2014)
			Flooding			Vegetative growth↓ (Du *et al*., 2014)
		Tobacco	Salt	Signaling	Overexpressing *TERF1* gene encoding ethylene response factor protein	Vegetative growth↑ (Tian *et al*., 2011)
		Tomato	Salt			Vegetative growth↑ (Huang *et al*., 2004)
		Rice	Drought			Vegetative growth↑ (Zhang *et al*., 2010)
		Rice	Cold		Overexpressing *TERF2* gene encoding ethylene response factor protein	Vegetative growth↑ (Du *et al*., 2014)
Gibberellin	Loss‐of‐function	Rice	Cold	Biosynthesis	Deficient GA‐insensitive dwarf1 gene	Vegetative growth↑ (Tanaka *et al*., 2006)
		Sunflower	Drought		Deficient GA‐insensitive *dwarf2* gene encoding ent‐kaurenoic acid oxidase	Vegetative growth↑ (Mariotti *et al*., 2022)
		Tomato	Drought		Deficient *gid1* or *gid2* gene	Vegetative growth↑ (Omena‐Garcia *et al*., 2019)
		Maize	Drought		Deficient *ks3‐1* gene encoding kaurene synthase	Vegetative growth↑ (Wu *et al*., 2023)
	Gain‐of‐function	Rice	Salt	Biosynthesis	Overexpressing *GA2ox5* gene encoding GA 2‐oxidase	Vegetative growth↑ (Wu *et al*., 2023)
Jasmonic acid	Loss‐of‐function	Tomato	Salt	Biosynthesis	Deficient *def‐1* gene encoding defenseless‐1	Vegetative growth↑ (Abouelsaad & Renault, 2018)
		Tomato	Cadmium		Deficient spr2 gene encoding chloroplast fatty acid desaturase	Vegetative growth↓ (Zhao *et al*., 2016)
		Maize	Salt		Deficient *opr7opr8* gene encoding oxo‐phytodienoic acid reductases	Seedling growth↓ (Ahmad *et al*., 2019)
	Gain‐of‐function	Rice	Drought	Metabolism	Overexpressing *JMT* gene encoding jasmonic acid carboxyl methyltransferase	Reproductive growth↓, grain yield↓ (Kim *et al*., 2009)
Salicylic acid	Loss‐of‐function	Tobacco	Drought	Signaling	Overexpressing SABP gene encoding SA‐binding protein 2	Vegetative growth↑ (Li *et al*., 2019)
Strigolactone	Loss‐of‐function	Barley	Drought	Signaling	Deficient *hvd14.d* gene encoding α/β hydrolase	Vegetative growth↓ (Marzec *et al*., 2020)
		Tomato	Drought		Downregulating *CCD7* gene encoding carotenoid‐cleavage dioxygenases	Vegetative growth↓ (Visentin *et al*., 2016)

Gene name in *italic*.

Despite growing evidence supporting the role of hormone signaling mutants in stress tolerance, their practical application remains limited (Li & Chen, 2023). Several challenges hinder implementation. Most mutations in hormone pathways arise from random variations induced by physical or chemical mutagenesis rather than natural biological processes such as domestication or diversification (Bado *et al*., 2015). Hormone signaling and stress response networks involve numerous genes with complex interactions, making it difficult to modify a single gene without unintended negative consequences. Even with a well‐characterized target protein, mutational scanning is necessary to evaluate effects on activity, stability, dosage and potential pleiotropic outcomes (Soskine & Tawfik, 2010). New breeding techniques are rapidly enabling precise modulation of plant hormone pathways; these tools include allele discovery, precision allele editing, dosage control strategies, promoter discovery and engineering and tissue‐specific editing (Jayakodi *et al*., 2025).

### The roles of exogenous hormones in crop stress regulation

Exogenous bioactive compounds, whether naturally occurring or chemically synthesized, have been used to help alleviate plant stress (Fig. 1b). These compounds can be applied directly or induced through microbial inoculation with uptake via multiple pathways, including passive diffusion, active transport through plasma membrane transporters, endocytosis, stomata and via symbiotic mechanisms like nodulation and mycorrhiza. Box 1 summarizes classical hormones and analogs involved in plant growth and development, while Table 2 illustrates how the exogenous application of these compounds, including both agonists and antagonists, enhances stress tolerance across different crop species.

**Table 2:** The application of natural‐occurring plant hormones and their synthetic analogs and their influence on crop stress responses.

Plant hormone	Chemical nature	Compound name	Function	Application method	Treated crop	Stress condition	ROS	Osmotic substance	Mitigated phenotype
Abscisic acid	Natural occurring	(+)‐*cis*,*trans*‐ABA (S‐ABA)	Signaling	Foliar spray pretreatment	Maize	Drought	↑	Proline↑, MDA↓	Germination↑, seedling growth↑ (Yao *et al*., 2019)
				Foliar spray	Rice	Salt	↓	Na^+^/K^+^↓	Vegetative growth↑ (Jiang *et al*., 2024)
	Synthetic agonist	Quinabactin	Signaling	Foliar spray pretreatment	Soybean, barley, maize	Drought	–	–	Vegetative growth↑ (Okamoto *et al*., 2013)
		Opabactin		Foliar spray pretreatment	Wheat	Drought	–	–	Vegetative growth↑ (Vaidya *et al*., 2019)
Auxin	Natural occurring	Indole‐3‐acetic acid (IAA)	Signaling	Foliar spray pretreatment	Rice	Drought and heat	↓	–	Grain yield↑ (Sharma *et al*., 2018)
	Synthetic antagonist	Naphthaleneacetic acid (NAA)	Signaling	Foliar spray	Chufa	Alkaline	↓	Na^+^/K^+^↓, MDA↓	Seedling growth↑ (Ullah *et al*., 2022)
				Soaking pretreatment	Pea	Drought	↓	MDA↓, EL↓	Seedling growth↑ (Xing *et al*., 2016b)
		2,4‐Dichlorophenoxyacetic acid (2,4‐D)		Soaking pretreatment	Wheat	Salt	↓	Na^+^/K^+^↓, MDA↓, EL↓	Seedling growth↑ (Mohsin *et al*., 2020)
Brassinosteroid	Natural occurring	24‐Epibrassinolide (EBL)	Signaling	Foliar spray	Wheat	Drought	↓	Proline↓, MDA↓, EL↓	Vegetative growth↑, grain yield↑ (I. Khan *et al*., 2021)
				Foliar spray pretreatment	Perennial Ryegrass	Salt	–	Na^+^/K^+^↓, Proline↑	Vegetative growth↑ (Wu *et al*., 2017)
	Synthetic agonist	BB16		Foliar spray pretreatment	Strawberry	Salt, drought	–	–	Vegetative growth↑, fruit yield↑, fruit quality↑ (Furio *et al*., 2022)
Cytokinin	Synthetic agonist	6‐Benzyladenine (6‐BA)	Signaling	Foliar spray	Maize	Flooding	↓	MDA↓, EL↓	Seedling growth↑ (Wang *et al*., 2021)
				Foliar spray	Winter wheat	Heat	–	–	Reproductive growth↑ (Yang *et al*., 2016)
		Kinetin		Foliar spray	Common sage	Salt	–	Na^+^/K^+^↓	Vegetative growth↑ (Tounekti *et al*., 2011)
		N‐(2‐chloro‐4‐pyridyl)‐N′‐phenylurea (CPPU)		Foliar spray	Rice	Drought	–	–	Vegetative growth↑ (Gujjar *et al*., 2020)
				Foliar spray pretreatment	Rice	Salt	–	Proline↑	Vegetative growth↑, grain yield↑ (Gashaw *et al*., 2014)
	Synthetic antagonist	PI‐55	Signaling	Seed treatment	*Bulbine natalensis* Baker *and Rumex crispus* L.	Cadmium	–	–	Seedling growth↑ (Gemrotová *et al*., 2013)
		2‐Chloro‐6‐(3‐methoxyphenyl)aminopurine (INCYDE)	Metabolism						
Ethylene	Synthetic agonist	Ethephon	Signaling	Foliar spray	Mustard	Salt	↓	Proline↑	Vegetative growth↑ (Iqbal *et al*., 2017)
	Synthetic antagonist	Aminoethoxyvinylglycine (AVG)	Biosynthesis	Foliar spray pretreatment	Cotton	Flooding	–	–	Vegetative growth↑, fruit yield↑ (Najeeb *et al*., 2015)
				Dipping pretreatment	Persimmon	Cold storage			Fruit quality↑ (Win *et al*., 2021)
		1‐Methylcyclopropene (1‐MCP)	Signaling	Irrigation	Rice	Salt	↓	Proline↓, MDA↓	Vegetative growth↑, grain yield↑ (Hussain *et al*., 2019)
Gibberellin	Natural occurring	GA3	Signaling	Foliar spray	Mustard	Salt	–	Na^+^/K^+^↓, MDA↓, EL↓	Vegetative growth↑ (Siddiqui *et al*., 2008)
				Seed pre‐soaking	Wheat	Salt	–	Na^+^/K^+^↓	Vegetative growth↑, grain yield↑ (Iqbal & Ashraf, 2013)
	Synthetic antagonist	Chlormequat chloride (CCC)	Biosynthesis	Seed treatment	Rice	Salt	–	–	Grain yield↑ (Gurmani *et al*., 2011)
		Mepiquat chloride (MC)		Seed priming	Cotton	Salt	–	Na^+^/K^+^↓	Seedling growth↑ (N. Wang *et al*., 2019)
		Flurprimidol		Soil drench pretreatment	Red Firespike	Drought	–	–	Vegetative growth↑ (Rezazadeh *et al*., 2016)
		Trinexapac‐ethyl		Foliar spray pretreatment	Perennial ryegrass	Drought	–	Proline↑, MDA↓, EL↓	Vegetative growth↑ (Sheikh Mohammadi *et al*., 2017)
				Foliar spray pretreatment	Kentucky bluegrass	Drought	–	EL↓	Vegetative growth↑ (Krishnan & Merewitz, 2015)
		Paclobutrazol (PBZ)		Foliar spray or soil drench pretreatment	Pomegranate	Cold	–	Proline↓, EL↓	Vegetative growth↑ (Moradi *et al*., 2017)
				Foliar spray	Wheat	Heat stress due to late Sow	↓	MDA↓, EL↓	Grain yield↑ (Nagar *et al*., 2021)
				Foliar spray	Strawberry	Drought	↓	Proline↓	Vegetative growth↑, fruit yield↑ (Saleem *et al*., 2024)
Jasmonic acid	Agonist	Methyl jasmonate (MeJA)	Signaling	Soaking	Black locust	Salt	↓	Na^+^↓, MDA↓	Vegetative growth↑ (Jiang *et al*., 2016)
				Vapor incubation pretreatment	Peach	Cold storage	↓	Na^+^↓, MDA↓	Postharvest fruit quality↑ (Jin *et al*., 2013)
Salicylic acid	Natural occurring	2‐Hydroxybenzoic acid	Signaling	Foliar spray	Maize	Drought	↓	MDA↓	Vegetative growth↑ (Saruhan *et al*., 2012)
				Foliar spray	Mustard	Salt	↓	Na^+^↓	Vegetative growth↑ (Nazara *et al*., 2015)
		Acetylsalicylic acid (ASA)		Seed soaking pretreatment and root treatment	Maize	Cold	↓	MDA↓	Seedling growth↑ (Wang *et al*., 2012)
Strigolactone	Agonist	GR24	Signaling	Foliar spray pretreatment	Cucumber	Salt	↓	Proline↑, EL↓, Na^+^/K^+^↓	Seedling growth↑ (Zhang *et al*., 2022)
				Root treatment	Barley	Cadmium	↓	MDA↓	Vegetative growth↑ (Qiu *et al*., 2021)
				Foliar spray	Maize	Drought		MDA↓	Vegetative growth↑, grain yield↑ (Luqman *et al*., 2023)
				Foliar spray	Apple	Saline‐alkali	↓	Na^+^/K^+^↓	Seedling growth↑ (C. Ma *et al*., 2022)
				Dipping pretreatment	Sweet orange	Cold storage	↓	MDA↓	Fruit quality↑ (Q. Ma *et al*., 2022)
				Foliar spray pretreatment	Cotton	Salt	↓	Proline↓, MDA↓	Seedling growth↑ (Song *et al*., 2023)

–, not reported; EL, electrolyte leakage; MDA, malondialdehyde; Na+, sodium ions; ROS, reactive oxygen species, including hydrogen peroxide (H_2_O_2_), superoxide radicals (O_2_^−^) and hydroxyl free radical (OH^−^).

Box 1 **Glossary of terms (in the context of this review)**

Active form: A hormone in an active form that is able to cause a specific effect.

Agonist: A hormone analog that mimics the mode of action of a naturally occurring hormone.

Analog: A small molecule whose physical structure is similar to a naturally occurring hormone.

Antagonist: A hormone analog that blocks the mode of action of a naturally occurring hormone.

Inactive form: A hormone in an inactive form that fails to cause a specific effect.

Precursor: The starting compound that is used for hormone biosynthesis.

Isomer: molecules that share a chemical formula, but not the same structure or orientation in space.

Naturally occurring auxins, including indole‐3‐acetic acid (IAA), 4‐chloroindole‐3‐acetic acid, indole‐3‐butyric acid (IBA) and phenylacetic acid (PAA), have been utilized to manipulate plant stress responses examples of which included the exogenous application of IAA improved grain yield in rice under drought and heat stress by enhancing pollen viability and spikelet fertility (Sharma *et al*., 2018) and enhanced protective mechanisms and resilience in vegetative growth under salinity stress of rapeseed seeds soaked in IBA (Li *et al*., 2024). While synthetic and natural auxins are primarily used for growth promotion or inhibition, auxin also plays a crucial role in regulating crop stress responses (Weijers *et al*., 2018). For example, the application of naphthaleneacetic acid (NAA) induces early IAA‐dependent accumulation of H_2_O_2_, enhancing antioxidant capacity and improving drought stress tolerance in soybean seedlings (Xing *et al*., 2016a).

Abs*cis*ic acid occurs as the (+)‐*cis*,*trans*‐isomer (S‐ABA), a chiral structure that is challenging to synthesize and is commercially produced through the fermentation of phytopathogenic fungi (Rademacher, 2015). The application of S‐ABA has been shown to enhance stress tolerance in various crops. In salt‐treated rice, S‐ABA improved photosynthesis by reducing Na^+^ accumulation in leaves and increasing the activity of antioxidant enzymes (Jiang *et al*., 2024). Similar stress‐alleviating effects have been observed in maize and apple under drought conditions (Tworkoski *et al*., 2011; Yao *et al*., 2019; Qiao *et al*., 2023). Additionally, synthetic receptor agonists of abscisic acid, such as pyrabactin analogs (e.g. quinabactin and opabactin), have been developed to selectively control stomatal conductance, offering potential applications for reducing water loss during drought stress (Okamoto *et al*., 2013; Liu *et al*., 2023).

Brassinosteroids include *c*. 70 polyhydroxylated sterol derivatives (Oklestkova *et al*., 2015). Despite significant efforts to chemically synthesize brassinosteroids, only two brassinosteroid analogs, EBL and BB16, are widely used in agriculture (Oklestkova *et al*., 2015). Foliar applications of both EBL and BB16 have been shown to enhance fruit production and improve fruit quality in strawberry plants subjected to salt and water stress (Furio *et al*., 2022) and have shown stress protective effects on rice and lettuce (Núñez Vázquez *et al*., 2013; Serna *et al*., 2015; Reyes Guerrero *et al*., 2017).

Natural cytokinins are N^6^‐substituted adenine derivatives, including compounds such as N,N′‐diphenylurea, N^6^‐[(3‐methylbut‐2‐en‐1‐yl)amino]purine (iP), zeatin, Z‐9‐riboside and meta‐Topolin, all of which are isolated from plant materials (Kamínek, 2015). While many synthetic 6‐benzyladenine (6‐BA)‐containing compounds with CK‐like bioactivities have been reported, only a few are commercially available due to challenges in achieving efficient synthesis (Rademacher, 2015). Exogenous application of 6‐BA has been shown to alleviate waterlogging‐induced damage in maize by activating the ROS scavenging system and promoting plant growth (Wang *et al*., 2021). Additionally, two novel cytokine derivatives, PI‐55 and INCYDE, have been developed to block cytokine signaling and inhibit cytokine degradation by targeting cytokine oxidase/dehydrogenase. These treatments prolong cytokinin activity in plants and positively affect shoot and root growth, as well as fresh weight, in medicinal plant seedlings grown in the presence of cadmium (Gemrotová *et al*., 2013).

Ethylene applications, inhibitors or antagonists are commonly used to promote rapid and uniform ripening in fruits (Brecht, 2019) but also have effects on plant stress responses. Mustard plants treated with ethephon, an ethylene agonist, under salt stress showed a significant reduction in oxidative damage and an enhancement in photosynthetic nitrogen use efficiency (Iqbal *et al*., 2017). Aminoethoxyvinylglycine (AVG) and 1‐methylcyclopropene (1‐MCP) are two effective ethylene antagonists that inhibit ethylene biosynthesis and signaling pathways respectively. Treatments with AVG and 1‐MCP prevented weight loss and reduced the decline in soluble solids content during cold storage of persimmon fruit by minimizing the incidence and severity of peel blackening and softening (Win *et al*., 2021). AVG and 1‐MCP also function as stress protectors during the growth period of crops such as rice, cotton and apple (Djanaguiraman *et al*., 2011; Chen *et al*., 2014; Mohammed *et al*., 2015).

Nearly 140 different gibberellins have been identified in higher plants and fungi. Commercially, gibberellins are typically obtained through the fermentation of phytopathogenic fungi, with GA_3_ being the most widely produced form, followed by GA_4_ and GA_7_ (Rademacher, 2015). The application of GA_3_ has been shown to improve grain yield in salt‐stressed wheat, with the effect being particularly pronounced in salt‐sensitive cultivars (Iqbal & Ashraf, 2013). Several gibberellin biosynthesis inhibitors act as growth retardants by reducing longitudinal shoot growth, resulting in a more compact plant architecture that lowers the risk of lodging and improves tolerance to various abiotic stresses. Strawberries treated with the gibberellin inhibitor Paclobutrazol (PBZ) under drought conditions exhibited enhanced enzymatic and non‐enzymatic antioxidant activities, increased relative water content and improved photosynthetic rate, resulting in higher fruit yield (Saleem *et al*., 2024).

Among jasmonates, (+)‐7‐iso‐JA‐L‐isoleucine (JA‐Ile), a stereoisomer conjugated with the amino acid isoleucine, has been recognized for its bioactivity in plants upon exogenous application (Yan *et al*., 2016). The oxidative damage caused by salt stress in black locust was alleviated by the exogenous application of Methyl jasmonate (MeJA), which significantly enhanced the activities of antioxidant enzymes (Jiang *et al*., 2016). Crops such as beans, cauliflower and okra have improved resistance to various stresses through the application of MeJA (Wu *et al*., 2012; Mohi‐Ud‐Din *et al*., 2021; Wang *et al*., 2023a, 2023b).

Natural salicylic acid (SA) is primarily produced by plants, and its analogs are widely used as pharmaceuticals due to their anti‐inflammatory properties (Rosheen & Utreja, 2023). SA or acetylsalicylic acid (ASA) treatments, applied through seed soaking or root treatment, significantly enhanced the tolerance of maize seedlings and young plants to chilling stress (Wang *et al*., 2012). The stress‐tolerant properties of SA have been observed in crops like rice, tomato and sunflower (Jini & Joseph, 2017; Noreen *et al*., 2017; Khan *et al*., 2019; Naeem *et al*., 2020).

Two stereochemical families of strigolactones, namely (+)‐strigol and (−)‐orobanchol, are the most abundant in nature. Their complex structures make large‐scale production challenging. Many studies on synthetic strigolactone analogs with easier‐to‐synthesize structures have primarily focused on their herbicidal activity, while research on their role in abiotic stress response has been comparatively limited (Zwanenburg & Blanco‐Ania, 2018). GR24, the most widely used synthetic strigolactone, has protective effects against abiotic stresses in barley, orange, cucumber, maize and cotton (Qiu *et al*., 2021; Zhang *et al*., 2022; C. Ma *et al*., 2022; Q. Ma *et al*., 2022; Luqman *et al*., 2023; Song *et al*., 2023).

Although the commercialization outlook for hormone‐like molecules is promising their use in scalable agriculture remains limited. Several factors contribute to this: insufficient studies on a variety of crops, a lack of long‐term and detailed dose–response trials and a generally insufficient understanding of the metabolic processes involved. The relatively limited commercial use of natural and synthetic plant hormones to manage plant stress responses is also due to regulatory constraints since products that alter plant growth regulatory processes are generally classified as pesticides, which severely limits their commercial development (EPA, 2024).

### Biostimulants and their role in abiotic stress tolerance

Biostimulants have emerged as promising products to enhance crop resilience against abiotic stresses (Calvo *et al*., 2014; Franzoni *et al*., 2022). According to the International Standards Organization definition, biostimulants are ‘product(s) that contain substance(s), microorganism(s), or mixtures thereof, that, when applied to seeds, plants, the rhizosphere, soil, or other growth media, act to support a plant's natural nutrition processes independently of the biostimulant's nutrient content’ (ISO 8157:^2022^). Biostimulants can contain various natural compounds of microbial or non‐microbial origin, as well as beneficial microorganisms, including bacteria, fungi and yeasts. The most widely studied biostimulants include those derived from seaweed, humic substances, protein hydrolysates and living microbes. A recent meta‐analysis assessed the effectiveness of various biostimulants on crop yield improvement and estimated an increase of *c*. 18% (Li *et al*., 2022). Table 3 summarizes some examples of different biostimulants and their role in plant stress mitigation. Here, we highlight some of the studies that provide experimental evidence for a direct mechanistic effect and discuss the possible mode of action of the different compounds.

**Table 3:** Effect of the application of non‐microbial and microbial biostimulants on crop stress responses.

Biostimulant category	Natural source	Active ingredient	Application method	Treated crop	Stress condition	ROS	Osmotic substance	Mitigated phenotype
Seaweed extracts	*Ascophyllum nodosum*	IAA, GA, CK, phenols, biopolymers, sugars	Foliar spray	Tomato	Drought	–	Proline↑	Fruit yield↑, fruit quality↑ (Ahmed *et al*., 2022)
	*Sargassum spp*.	IAA, phenols, biopolymers, sugars	Foliar spray	Tomato	Salt	↓	Proline↑	Vegetative growth↑ (Sariñana‐Aldaco *et al*., 2022)
	*Sargassum wightii*	IAA, GA, CK, phenols, biopolymers, sugars	Foliar spray	Okra	Salt	↓	Na^+^↓	Vegetative growth↑, fruit yield↑, fruit quality↑ (Khan *et al*., 2022)
Garlic extract	*Allium sativum*	IAA, GA, ABA, Kinetin, ascorbic acid, sugars	Seed pretreatment	Broad bean	Drought	↓	MDA↓	Vegetative growth↑ (Kasim, 2017)
Carrot extract	*Daucus carota*	IAA, GA, ABA, CK, ascorbic acid, sugars						
Moringa extracts	Moringa oleifera	IAA, GB, CK, ABA, sugars, phenols, ascorbic acid	Foliar spray	Common bean	Salt, heat	↓	MDA↓	Green pod yield↑ (Latif & Mohamed, 2016)
Protein hydrolysate	Sugar cane molasses and yeast extract (*Saccharomyces cerevisiae*)	Glycine betaine, peptides, amino acids	Fertigation	Tomato	Drought	↓	–	Fruit yield↑ (Francesca *et al*., 2021)
	Pumpkin seeds	Amino acids, peptides	Foliar spray	Common bean	Salt	↓	Proline↓, MDA↓	Green pod yield↑, Vegetative growth↑ (Sitohy *et al*., 2020)
Humic acid	Leonardite	Humic acid	Soil treatment	Finger millet	Salt	↓	Proline↓, MDA↓	Vegetative growth↑ (Rakkammal *et al*., 2024)
	Non‐specified	Humic acid	Seed priming	Rice	Salt	↓	Na^+^↓, MDA↓, Proline↓	Vegetative growth↑ (Shukry *et al*., 2023)
	Organic matter	Humic acid	Soil treatment	Maize	Drought	–	Proline↑	Grain yield↑ (Chen *et al*., 2022b)
PGPB	*Azotobacter vinellandii* SRI Az3	IAA, GA	Root inoculation pretreatment	Rice	Drought	–	MDA↓, Proline↑	Vegetative growth↑ (Pradhan *et al*., 2018)
	*Bacillus amyloliquefaciens RWL‐1*	ABA	Root inoculation	Rice	Salt	–	Proline↑	Seedling growth↑ (Shahzad *et al*., 2017)
	*Bacillus cereus* SA1	IAA, GA	Root inoculation pretreatment	Tomato	Drought	–	K^+^↑	Vegetative growth↑ (Khan *et al*., 2020)
	*Bacillus* strains	IAA	Root inoculation pretreatment	Rice	Drought	–	Na^+^/K^+^↓	Seedling growth↑ (M. A. Khan *et al*., 2021)
	*Ensifer meliloti* RD64	IAA	Seed inoculation pretreatment	Alfalfa	Drought	‐	Proline↑	Vegetative growth↑ (I. Khan *et al*., 2021)
	*Enterobacter cloacae*	ACC deaminase	Seed pretreatment	Wheat	Salt, heavy metal	↓	MDA↓	Vegetative growth↑ (Singh *et al*., 2022)
	*Leclercia adecarboxylata* MO1	IAA	Root inoculation	Tomato	Salt	–	Proline↑	Vegetative growth↑ (Kang *et al*., 2019)
	*Pseudomonas azotoformans*	ET	Root inoculation pretreatment	Tomato	Salt		Proline↓	Vegetative growth↑ (Liu *et al*., 2021)
	*Pseudomonas fluorescens* G20‐18	CK	Root inoculation pretreatment	Tomato	Drought	–	–	Vegetative growth↑ (Mekureyaw *et al*., 2022)
	*Pseudomonas putida* H‐2‐3	GA	Root inoculation pretreatment	Soybean	Drought	↓	Na^+^↓	Vegetative growth↑ (Kang *et al*., 2014)
	*Pseudomonas sp*. UW4	ET	Root inoculation pretreatment	Tomato	Salt	–	–	Vegetative growth↑ (Orozco‐Mosqueda *et al*., 2019)
	*Streptomyces sp*., *Pseudomonas sp*.	Polysaccharide	Seed pretreatment	Wheat	Salt	–	Proline↓	Vegetative growth↑ (Thakur & Yadav, 2024)
AMF	*Rhizophagus irregularis*	IAA, CK, GA, ET	Root inoculation pretreatment	Black locust	Drought	↓	MDA↓	Vegetative growth↑ (He *et al*., 2017)
	*Funneliformis mosseae*	Biopolymers, chelating compounds	Seed treatment	Maize	Heat	↓	Proline↑	Vegetative growth↑ (Ye *et al*., 2019)
Endophytic fungi	*Paecilomyces formosus* LHL10, *Penicillium funiculosum* LHL06	Chelating compound	Root inoculation pretreatment	Soybean	Drought, heavy metal	↓	MDA↓	Vegetative growth↑ (Bilal *et al*., 2020)
	*Paecilomyces formosus* LHL10	GA, IAA	Root inoculation pretreatment	Cucumber	Salt	↓	MDA↓	Vegetative growth↑ (Latif Khan *et al*., 2012)
	*Trichoderma* spp.	GA, ABA, SA, IAA, CK	Root inoculation pretreatment	Wheat	Drought	↓	–	Vegetative growth↑ (Illescas *et al*., 2021)

−, not reported; ACC, 1‐aminocyclopropane‐1‐carboxylate; AMF, arbuscular mycorrhizal fungi; Cl^−^, chloride ions; EL, electrolyte leakage; MDA, malondialdehyde; Na^+^, sodium ions; PGPB, plant growth‐promoting bacteria; ROS, reactive oxygen species, including hydrogen peroxide (H_2_O_2_), superoxide radicals (O^2−^) and hydroxyl free radical (OH^−^).

Species are indicated in *italic*.

#### Nonmicrobial biostimulants

Seaweed‐derived biostimulants, like seaweed extracts (SE), contain a rich array of bioactive compounds, including phytohormones like auxins, CKs and GAs, vitamins, amino acids and polysaccharides, which contribute to their beneficial effects (Nanda *et al*., 2021). Tomato treated with SE improved fruit yield under salinity conditions (Hernández‐Herrera *et al*., 2022). The effect was partially attributed to the presence of phytohormones in the extract. The plant hormones auxin, GAs and CKs have been detected in SE and can persist in the final applied extract, although in variable concentrations (Sangha *et al*., 2014). For example, GAs and CKs have been observed to range from 0.3 to 4.7 μg g^−1^ and from 0.06 to 4.6 μg g^−1^ of seaweed dry weight, respectively, while auxin has been identified with concentrations between 0.01 and 12 μg g^−1^. SE has shown the capability to alter the endogenous levels of plant hormones (Deolu‐Ajayi *et al*., 2022). SE from *Kappaphycus alvarezii* significantly increased endogenous ABA and CKs concentrations in durum wheat in both non‐stressed and drought conditions (Patel *et al*., 2018). The observed increase of maize tolerance to drought stress upon SE application was attributed to both the increase of polyamines and to the stimulation of IAA and GA endogenous production (Li *et al*., 2018).

Many SE biostimulants enhance stress resistance by boosting the plant's antioxidant defenses, by triggering various physiological and biochemical pathways, such as the synthesis of osmoprotectants and thus reducing oxidative stress caused by abiotic factors (A. H. Ali *et al*., 2022). The stress‐protective effect of SE against salt stress was associated with the presence of phenolic compounds that can both act as ROS scavengers and chelate toxic ions (Carillo *et al*., 2020). Antioxidant and stress‐protective effects have also been attributed to the presence of non‐structural carbohydrates and biopolymers (Elansary *et al*., 2016). Algal polysaccharides such as ulvans, alginates and fucans can act as elicitors by activating stress‐related pathways in plants, thus resulting in hormone‐like effects (Chanda *et al*., 2019). In‐soil application of algal polysaccharides improved salt stress tolerance of wheat, as a function of the molecular weight and the sulfate content of the polymers (Bouteraa *et al*., 2022), and induced an antioxidant response by modulating Na+ uptake and mobilization within the crop and by regulating the expression of Na+ transporters (Zou *et al*., 2019). Finally, the presence of polyamines in SE can have osmoregulatory effects and protect crops in water‐deficient conditions (Rugiu *et al*., 2020).

Plant extracts (PE) are usually concentrated liquids or powders extracted from various plant species. Some PE may contain natural plant growth regulators, such as auxins, CKs and GAs, which are an inevitable consequence of their plant origin. PE can also induce hormone production. The application of PE on common flax increased the endogenous levels of GA3 and IAA both at the shooting and rooting stages (Oguz, 2024). Other bioactive compounds present in PE, such as sugars, can up‐regulate growth‐related genes while downregulating stress‐related ones, and thus alleviate salt stress in rice (Ho *et al*., 2001). Extracts obtained from Moringa oleifera protected common bean from salinity and heat stress (Latif & Mohamed, 2016). PE also contains a variety of other bioactive compounds, including alkaloids, flavonoids, phenols, terpenoids and essential oils, which contribute to their biological properties (Naboulsi *et al*., 2018; Kowalczewski & Zembrzuska, 2023).

Fulvic acids (FA), humic acids (HA) and humates are highly recalcitrant organic substances derived from the decomposition of plant and animal matter (Canellas *et al*., 2015). Foliar application of HA improved drought resistance of wheat by increasing its antioxidant response (M. A. Khan *et al*., 2021). The stress‐protective effects of HA and humates were attributed to a hormone‐like activity leading to the activation of stress‐dependent pathways (Olaetxea *et al*., 2018; Canellas *et al*., 2024). HA can help regulate osmotic pressure within plant cells, aiding in the maintenance of turgor pressure and overall cellular function during water stress and improving grain yield by 16% in two different maize genotypes exposed to drought stress (Chen *et al*., 2022b). These HA‐induced stress‐protective effects were observed along with shifts in endogenous hormonal levels, including an increase of IAA and a decrease of ABA concentrations. The growth‐stimulating effect of HA on wheat was also related to the modulation of endogenous hormonal pathways, as gene analysis revealed the HA‐induced up‐regulation of genes involved in the biosynthesis of auxin and CKs (Rathor *et al*., 2024).

Protein hydrolysates (PH) are derived from the enzymatic or chemical hydrolysis of proteins from various sources, including animal byproducts, plant biomass and microbial fermentations of various carbon compounds (Colla *et al*., 2014; Gao *et al*., 2021). PH are usually rich in amino acids and peptides, which act as biostimulants through both soil and foliar application (Colla *et al*., 2015a). PH stimulate root and shoot development and promote overall plant vigor, which is crucial for coping with abiotic stressors like drought and salinity (Casadesús *et al*., 2020). In some cases, the presence of tryptophan – an auxin precursor – in PH has been attributed to the stimulation of auxin‐like responses resulting in the promotion of seed germination and plant growth of pea (*Pisum sativum* L.) (Colla *et al*., 2017). Root growth promotion by PH can enhance the plant's ability to access water and nutrients, improving resilience against drought and nutrient deficiencies (Casadesús *et al*., 2020; Ceccarelli *et al*., 2021). The PH treatment of water‐stressed tomato plants was associated with increased GA1, GA3 and IAA endogenous levels and decreased ABA concentrations (Casadesús *et al*., 2019). Metabolic analyses suggested that PH of various origins alter both phytohormone profiles and fatty acid metabolism of the treated plants (Ceccarelli *et al*., 2021).

PH can influence plant stress response by mechanisms not obviously related to hormone metabolism. PH improve nutrient solubility and availability, facilitating the uptake of essential nutrients by plants during stress conditions (Rouphael *et al*., 2020). Seed treatment with PH‐protected tomato plants from heat and drought‐induced damage by preserving yield and quality traits (Francesca *et al*., 2022). These protective effects were observed along with an increase in antioxidant content within the plant (W. Wang *et al*., 2022). The application of PH to maize seedlings in a hydroponic system improved the plant's tolerance to salt, nutrient deficiency and hypoxia stress conditions (Trevisan *et al*., 2019). The effects were traced back to a PH‐related modulation of nitrate transporters and ROS gene expression. PH function can also vary depending on the source of hydrolysates. For example, 2 out of 11 PH tested as seed primers resulted in salt stress‐alleviating properties (Sorrentino *et al*., 2021). By contrast, different types of PH had similar but crop‐dependent salt stress alleviating effects (Zuluaga *et al*., 2023).

#### Microbial biostimulants

Interest in the use of plant microbial inoculation to enhance crop stress tolerance is founded on the observation that the imposition of abiotic stress often results in functional changes to the plant microbiome that can enhance plant stress tolerance, and from the observation that stress‐tolerant species are often associated with specific microbial partners critical for the tolerance of those species to the stress (Fitzpatrick *et al*., 2017; Timm *et al*., 2018). Microbiome shifts under plant stress vary with the specific abiotic stress conditions, with some ‘core species’ remaining preserved, indicating a strict relationship between the plant and its associated bacteria (Timm *et al*., 2018). Drought conditions enriched Actinobacteria over other Bacteroidetes and Proteobacteria in bulk soil, due to the higher resistance of Actinobacteria to drought conditions. The inoculation of stress‐tolerant microbes to the plant has also been shown to increase abiotic stress tolerance (Enebe & Babalola, 2018). Microbial biostimulants, including both bacteria and fungi, have the potential to synthesize IAA, as predicted from the analysis of 7282 prokaryotic genomes and empirical evidence (Keswani *et al*., 2020) and the beneficial effect of microbial biostimulants has often been attributed to the production of phytohormones (S. Ali *et al*., 2022; O. Ali *et al*., 2022; A. H. Ali *et al*., 2022). It has been widely hypothesized that treating plants with stress‐tolerant‐plant‐beneficial microbes can help restore stress‐induced microbiome imbalances and contribute to stress alleviation. Some examples of microbial biostimulants, including bacterial and fungal formulations, are reported in Table 3.

The *in‐vitro* inoculation of rice seedlings with the rhizobacterium *Bacillus altitudinis* resulted in a phenotypic modification of root architecture that was attributed to a change in IAA endogenous levels within the root and to the genetic modulation of auxin‐responsive genes involved in root formation (Ambreetha *et al*., 2018). A similar increase in endogenous IAA was observed upon the inoculation of wheat with rhizobacteria, including *Dietzia natronolimnaea*, *Arthrobacter protophormiae* and *Bacillus subtilis*, resulting in an increased tolerance against both drought and salinity (Barnawal *et al*., 2017). A bacterial consortium composed of *Staphylococcus epidermidis* CK9 strain and *Bacillus australimaris* CK11 inoculated on Arabian balsam tree (*Commiphora gileadensis*), improved tolerance to both salinity and drought stress and decreased endogenous levels of ABA and JA, while stimulating SA accumulation (Jan *et al*., 2024). Further investigations suggested that microbial biostimulants can influence the expression of the *TaCTR1* gene, involved in plant response to various stress types (Bi *et al*., 2010). The inoculation of *Bacillus casamancensis* MKS‐6 and *Bacillus* sp. MRD‐17 in mustard counteracted drought stress by influencing both plant endogenous hormonal levels – including ABA and GAs – and ABA‐independent signaling, by downregulating *BjDREB1_2* and *BjDREB2* transcription factors (Nivetha *et al*., 2024). In this case, the presence of bacterial phytohormones has been shown to also counteract the growth‐inhibition effect of abiotic stress. In durum wheat exposed to drought and salinity stress in a greenhouse experiment, plants treated with a microbial consortium were more tolerant compared to untreated plants (Yaghoubi Khanghahi *et al*., 2022). Results suggested that microbial biostimulant inoculation also improved the grain quality of wheat, enhancing protein, sugars and lipid content under stress.

Fungal biostimulants have also been effective in plant stress mitigation. Seed pretreatment with the fungal strain *Trichoderma lixii* improved plant and root development and osmolyte accumulation of maize exposed to salt stress (Pehlivan *et al*., 2017). It was proposed that *Trichoderma* can both trigger various stress defense responses in plants and stimulate plant rooting and growth parameters via the production of IAA, as well as adsorb and chelate toxic ions via the production of siderophores, including excess Na + as the possible consequence of altered ion mobility (Colla *et al*., 2015b; Yadav *et al*., 2024).

In addition to the direct effects of microbial biostimulants on phytohormone production, a diversity of microbial‐dependent stress‐protective mechanisms has been proposed (Table 3). Microbial strains producing 1‐aminocyclopropane‐1‐carboxylate (ACC) deaminase can lower ethylene levels and improve plant responses to various types of stresses (Glick, 2014; Jha *et al*., 2021). The multiple inoculation of basil (*Ocimum sanctum* L.) with ACC deaminase‐producing microbes resulted in lower levels of ACC, increased yield and phenolic content and consequently alleviated cold‐dependent growth inhibition (Singh *et al*., 2020). Similarly, the inoculation of groundnut (*Arachis hypogea*) exposed to saline stress conditions with ACC‐producing *Pseudomonas fluorescens* increased yield (Saravanakumar & Samiyappan, 2007).

The difficulty in obtaining effective root colonization and persistence in soil and plant over time is a challenging issue when assessing the effectiveness of MBS and plant‐beneficial microbes (Romano *et al*., 2020). Efficient delivery systems, together with improved understanding of plant–microbe interaction, can help reduce yield losses due to abiotic stress and improve the efficacy of MBS in the target plants. The integration of MBS in agricultural practices presents a promising strategy for enhancing plant resilience to abiotic stress, offering potential benefits for crop quality and for agricultural sustainability in changing environmental conditions.

## Effects of the exogenous application of plant hormones, plant nutrients and biostimulants on endogenous hormone levels and plant response

To ascertain if the application of biostimulants of microbial products results in non‐natural concentrations of plant hormones, a survey of concentrations observed in nature is instructive. The extent to which *in vivo* plant hormone levels can change under stress and non‐stress conditions, during plant development and in response to the application of hormones, nutrients and biostimulants under different agronomic treatments and in different plant species is provided in Table S1. Accurately measuring the effects of various stress mitigation strategies on *in vivo* plant hormone activity will be critical to optimizing the use of exogenous hormones and biostimulants as stress mitigation strategies. Measurement of plant hormones *in vivo* is, however, complicated by detection constraints and low expression levels with strong temporal and spatial variation (Jones, 2016).

A wealth of literature demonstrates that micro‐ and macro‐nutrients can regulate hormone biosynthesis, signaling and transport (Rubio *et al*., 2009; Jia *et al*., 2022), and nutrient deficiencies can alter the plant's hormonal response, triggering hormonal stress‐like responses (Wittenmayer & Merbach, 2005). The application of silicon, for example, has been observed to both correct nutrient deficiency, alter plant hormone levels and enhance plant stress tolerance (Hosseini *et al*., 2017; Réthoré *et al*., 2020). Similarly, biostimulants such as humates have been observed to trigger hormone‐mediated stress‐alleviating effects under nutrient deficient conditions (Othibeng *et al*., 2021), suggesting that the stress‐related response mechanisms, plant nutrient status and related metabolic networks impacting hormonal pathways are often intertwined and not easy to distinguish (Table S1). Stress events, including nutrient deficiency or excess, also result in direct changes to cellular hormone levels depending on growth conditions and the species analyzed. Moreover, in response to stress, hormone levels are observed to rise or decrease with effects on primary and secondary metabolism and growth, making it difficult to disentangle the impact of plant hormone levels from other metabolic changes (Table S1).

Overall, the level of alteration in endogenous hormone concentrations in response to exogenous application of bioactive compounds, such as biostimulants, nutrients, plant hormones or microbes, is usually small compared to stress imposition, which can often result in a more consistent change in endogenous hormonal levels, reflecting the role of plant hormones in stress response strategies.

## Summary and the implications of regulatory constraints on the development and use of biostimulants, microbials and plant hormones to enhance climate resilience

The increased occurrence and severity of extreme weather events is among the greatest threats to agricultural productivity globally. Extreme weather events not only reduce crop productivity by compromising crop photosynthesis, metabolism and growth, they also disrupt normal agronomic practices and hence compromise farming efficiency and profitability. Mitigating unpredictable and highly localized extreme weather events will require the development of technologies that can be rapidly and locally implemented to enhance the resilience of the crop to the impending stress. These ‘rapid response’ technologies will supplement longer‐term breeding and cropping system strategies that aim to enhance the natural resilience of the crop and the cropping system. Biostimulants represent powerful tools to achieve this goal.

Plant tolerance to climate stress is largely mediated through plant sensing mechanisms and the regulation of plant hormone pathways. Plant breeding, agronomic inputs (nutrients and water), plant hormones and biostimulants have all been used to improve plant stress tolerance often mediated through interactions with natural plant hormone networks. Progress in the utilization of biostimulants, microbials and plant hormones to address the challenges of climate stress is constrained by regulatory frameworks that in many parts of the world classify all products that contain or explicitly modulate plant growth regulator pathways as pesticides. The classification of a product as a pesticide is associated with very strict safety requirements that lead to a substantial financial burden on product registration and commercial use; therefore, limiting the development and use of such products in agriculture. The requirements imposed on products containing plant hormones or products that modulate plant growth and development derive from US and EU law developed in the 1970s in response to the widespread use of hormones or hormone disruptors as herbicides. The following statements are illustrative of prevailing global regulations governing the use of pesticides in agriculture according to the US Federal Insecticide, Fungicide and Rodenticide Act (FIFRA):

US_FIFRA: *With certain exceptions, a pesticide is any substance or mixture of substances intended for preventing, destroying, repelling, or mitigating any pest, or intended for use as a plant regulator.*

The term ‘*plant regulator*’ is further defined as: ‘any substance or mixture of substances intended, through physiological action, for accelerating or retarding the rate of growth or rate of maturation or altering the behavior of plants.’ It is specifically stated in Environmental Protection Agency (EPA) rulings that all known plant hormones are considered pesticides and that products that contain known plant hormones or that explicitly claim to act through the modification of these pathways would be deemed pesticidal.

The breadth of this regulatory judgment is problematic as it implies that all products that alter plant growth are *pesticides* unless explicitly exempted through subsequent regulatory rulings. Interpreted literally, this definition would imply that plant breeding or the use of plant nutrients to improve plant growth and development could be regarded as pesticidal. Recognizing the undue burden this definition poses on normal agricultural practices such as fertilization, irrigation and soil amendments, the US EPA has created a positive list of exempt product classes specifying that ‘a product of any of the following types, intended only to aid the growth of desirable plants, is not a “plant regulator” under section 2(v) of FIFRA, and therefore is not a pesticide’ (FIFRA Amendment 40 CFR § 152.6). In 2024, the US proposed, through the introduction of a bill to the US House of Representatives, that biostimulants would obtain a similar exemption under FIFRA. In the EU, biostimulants are currently exempt from pesticide Regulation (EC) 1107/2009 according to Article 2(b) as they are classified as one category of fertilizing products (2019/1009, 2019).

While the proposed US biostimulant exemption and the existing EU biostimulant category appear to offer pathways for the use of these products to alleviate plant stress, the conflict with existing plant growth regulator definitions, and the evidence provided here that crop resilience is mediated through changes in the regulation of plant growth and development, represent a substantial scientific and marketing impediment to the full rational development and implementation of these promising tools (du Jardin *et al*., 2025). The inability of manufacturers of biostimulants, plant hormones or plant growth regulators to use these products to beneficially target plant regulatory networks, or beneficially modulate plant hormone pathways, or publicly claim such effects on labels or marketing materials, severely constrains progress. The implication that manufacturers of biostimulants must avoid the suggestion that enhanced plant stress tolerance is mediated by plant regulatory pathways is antithetical to the underlying science and inconsistent with natural resilience mechanisms where substantial fluctuations in internal plant regulatory pathways and hormone concentrations are a natural crop response to stress.

## Conclusions and future prospectives

Solutions will be needed for both the long‐term effects of climate change and the more immediate and critical impacts of increasingly frequent extreme weather events that cause profound agronomic disruption. While targeted breeding can provide robust solutions for long‐term climate threats, it requires an extended investment of time and money and must be replicated for each discrete cropping system. This process is inherently too slow to address the unpredictable and highly local extreme climate events.

Biostimulants, plant hormones and microbial inoculants have tremendous potential as tools that are more flexible and rapidly implementable alternatives to breeding. Coupling the application of biostimulants or plant hormones with weather prediction and just‐in‐time precision application has the potential to reduce the negative effects of abiotic stress on crop production while offering a highly tailored solution to local challenges.

The use of biostimulants, hormones or microbials and other products to address the threat of climate change, extreme weather and abiotic stress is novel and, as such, does not have a suitable enabling regulatory framework. The lack of globally accepted regulatory standards for the design and use of biostimulants has stifled their development, hindered their application and compromised grower acceptance. The current scientifically unsound and outdated legislative paradigm specifying ‘that any product acting directly upon plant growth processes’ is de facto ‘pesticidal’ represents the greatest barrier to the development and use of these products in agriculture. Given that most plant hormones, when present at concentrations found in nature, have been designated as safe, with no viable human toxicity nor environmental persistence, there appears to be no justification for limiting their use in agriculture when ‘used for the benefit of the target crop at naturally occurring concentrations’.

There is an immediate need to differentiate between products of negligible toxicity that are used solely for the benefit of the targeted crop (including plant growth regulators and biostimulants) and those products that are used solely to constrain or impede the growth of non‐beneficial plants (herbicides). Classification of products with dual‐use components according to the rate of use or intent of use of the product is easily implemented, with many examples of such frameworks already in place.

Regulatory clarity is necessary to foster innovation and enable the deployment of biostimulants to help reduce the negative impacts of climate change and extreme weather on crop production. These are powerful tools with tremendous potential to enhance crop productivity in the face of stress and hence improve the efficiency of use of inputs such as water, nutrients and pesticides.

## Competing interests

None declared.

## Disclaimer

The New Phytologist Foundation remains neutral with regard to jurisdictional claims in maps and in any institutional affiliations.

## Supporting information


**Table S1** Endogenous levels of auxin (IAA), abscisic acid (ABA), cytokinins (CK), gibberellins (GA), jasmonic acid (JA) and salicylic acid (SA) expressed as significant concentration increase or decrease under stress conditions and in response to application of exogenous compounds and treatments.
Please note: Wiley is not responsible for the content or functionality of any Supporting Information supplied by the authors. Any queries (other than missing material) should be directed to the *New Phytologist* Central Office.
