# Supplementary material for: The optimization of crop response to climatic stress through modulation of plant stress response mechanisms. Opportunities for biostimulants and plant hormones to meet climate challenges
Source: New Phytol. 2025 Nov 10;249(1):130–51. doi: 10.1111/nph.70701 (PMC12676087; doi:10.1111/nph.70701)
Supplement: Supplementary file 1 — Table S1 Endogenous levels of auxin (IAA), abscisic acid (ABA), cytokinins (CK), gibberellins (GA), jasmonic acid (JA) and salicylic acid (SA) expressed as significant concentration increase or decrease under stress conditions and in response to application of exogenous compounds and treatments. Please note: Wiley is not responsible for the content or functionality of any Supporting Information supplied by the authors. Any queries (other than missing material) should be directed to the New Phytologist Central Office. [file NPH-249-130-s001.pdf]

New Phytologist Supporting Information

The optimization of crop response to climatic stress through modulation of plant stress response mechanisms. Opportunities for biostimulants and plant hormones to meet climate challenges.

Jing Li[1]*, Giulia Forghieri[2]*, Danny Geelen[1], Patrick du Jardin[3], Patrick H. Brown[4]#

1 HortiCell, Department Plants and Crops, Faculty of Bioscience Engineering, Ghent University, Coupure Links 653, Ghent 9000, Belgium

2 CatMat lab, Department of Molecular Sciences and Nanosystems, Ca' Foscari University of Venice and Consortium INSTM UdR VE, via Torino 155, Venice 30172, Italy

3 Plant Sciences, Gembloux Agro-Bio Tech, University of Liège, Gembloux 4000, Belgium

4 Department of Plant Science, University of California Davis, One Shields Ave, Davis 95616, CA, USA

*These authors contributed equally.

#Corresponce: phbrown@ucdavis.edu

Article acceptance date: 9 October 2025

Supplementary Table 1. Endogenous levels of auxin (IAA), abscisic acid (ABA), cytokinins (CK), gibberellins (GA), jasmonic acid (JA) and salicylic acid (SA) expressed as significant concentration increase or decrease under stress conditions and in response to application of exogenous compounds and treatments, with respect to the control. Data are expressed as concentration per fresh or dry weight (d).

**Compound**[a]	**Experimental conditions**	**Stress**[b]	**Crop**	**Increase (+) decrease (–)**[c]						**Unit**	**Ref.**
				**IAA**	**ABA**	**CK**	**GA**	**JA**	**SA**		
None	Growth chamber	Salinity	Wheat	-0.1	+0.6	-0.02	-	-	-	µg/g	(Shakirova *et al.*, 2003)
None	Hydroponic system	Sulfur deficiency	Rice leaves	-	+24	-	-	+4.0	+1000	µg/g	(Réthoré *et al.*, 2020)
None	Growth chamber	Cold	Wheat spikelet	-8.0	+0.7	-	-7.0	-	-	ng/g	(Zhang *et al.*, 2019)
None	Growth chamber	Drought	Maize leaves	-3.0	+1.5	-3.5	-1.2	-	-	µg/g	(Wang *et al.*, 2008)
None	Greenhouse pot	Salinity	Soybean shoots	-	+1.0 (d)	-0.001 (d)	n	+0.02 (d)	+1.0 (d)	µg/g	(Hamayun *et al.*, 2010)
None	Growth chamber	Drought	Rice leaves	-1.5	-	-	-	+9.0	-	ng/g	(Du *et al.*, 2013)
		Cold		+3.0	-	-	-	+12	-		
		Heat		+2.0	-	-	-	-3.0	-		
Manganese	Greenhouse pots	Salinity	Rice shoot	+5.0	n	-	-	-	-	ng/g	(Nadeem *et al.*, 2020)
Zinc	Greenhouse pots	Salt	Rice shoot	+10	-5.0	-	-	-	-		
Copper sulfate	Hydroponic system	None	Maize leaves	+0.02	+0.008	+0.025	-	+0.05	-	nmol/g	(Reckova *et al.*, 2019)
Hydrogen sulphide	Growth chamber	Iron deficiency	Soybean leaves	+0.2 (d)	-0.6 (d)	n	+1.0 (d)	-1.7 (d)	+10 (d)	µg/g	(Chen *et al.*, 2020)

Iron	Growth chamber	Iron deficiency	Soybean leaves	+0.05 (d)	-0.4 (d)	+3.5 (d)	+4.0 (d)	-1.5 (d)	+12 (d)		
Molybdenum	Growth chamber	Cold	Wheat	-	+14	-	-	-	-	μg/g	(Sun *et al.*, 2009)
Phosphorous	Hydroponic system	Nutrient deficiency	Barley roots	+0.12	+0.001	-0.00005	+0.25	+3.0	+0.01	μg/g	(Nadira *et al.*, 2016)
Silicon	Hydroponic system	Salinity	Soybean	-	-0.8	-	-	-	-	μg/g	(Lee *et al.*, 2010)
Silicon	Growth chamber	Potassium deficiency and osmotic	Barley leaves	-	-100	-	-	-	-	pg/g	(Hosseini *et al.*, 2017)
SA	Growth chamber	Salinity	Wheat	+0.10	-0.2	+0.03	-	-	-	μg/g	(Shakirova *et al.*, 2003)
SA	Growth chamber	None	Wheat	+0.25	+0.15	n	-	-	-	μg/g	(Shakirova *et al.*, 2003)
GA_3	Greenhouse pot	Salinity	Soybean	-	-1.0 (d)	-	+0.01 (d)	+0.03 (d)	-0.005 (d)	μg/g	(Hamayun *et al.*, 2010)
IAA	Field	None	Fragrant Pear calyx tube	+0.028	+0.010	-	+0.20	-	-	μg/g	(Chen *et al.*, 2022a)
Naphthalene acetic acid	Growth chamber	Drought	Soybean	+400 (d)	+900 (d)	-	-	-	-	μg/g	(Xing *et al.*, 2016)
SA	Growth chamber	Salinity	Barley leaves	+3.8	-0.38	-	-	-0.03	-	nmol/g	(Torun *et al.*, 2022)
ABA	Greenhouse pot	Drought	Pea	+19.0	-1.5	-	+21.0	-	-	μg/g	(Latif, 2014)
GA_3	Field	Cold	Wheat	-	+0.08	-	-	-	-	μg/g	(Wang *et al.*, 2015)

Humic acid seed priming	Growth chamber	None	Rice shoots	-	-5.0	-	+6.0	-	-	ng/g	(Sheteiwy *et al.*, 2017)
Fulvic acid	Growth chamber	Heat	Soybean leaves	-	+0.05	-	-	-	-	µg/g	(Dinler *et al.*, 2016)
Humic acid	Field	Drought	Maize leaves	+20	-	-	-	-	-	nmol/g	(Chen *et al.*, 2022b)
Humic acid	Field	None	Mango	+1.15	-0.23	n	+2.50	-	-	µg/g	(El-Hoseiny *et al.*, 2020)
Fulvic acid	Field	None	Maize leaves	+0.06	-128	+18	+0.03	-	-	µmol/g	(Gao *et al.*, 2022)
			Maize roots	+0.03	-	+0.04	-	-	-		
Chitosan	Greenhouse pot	None	Basil	-	-0.02	-	-	-0.05	+0.21	µg/g	(Paulert *et al.*, 2021)
Algal extracts (*Sargassum tenerrimum*)	Greenhouse pot	None	Tomato leaves	-10	+30	n	-	-	+25	nmol/g	(Khedia *et al.*, 2020)
Algal extracts (*Ascophyllum nodosum*)	Greenhouse	None	Tomato	+28	-	+7.0	+5.0	-	-	µg/g	(Ali *et al.*, 2022)
Algal extracts (*Ascophyllum nodosum*)	Field	None	Hazelnut leaves	n	+2.5	-	+75	-	-	µg/g	(Cabo *et al.*, 2020)
Algal extracts (*Kappaphycus alvarezii*)	Open air pot	Drought	Wheat	+0.25	+0.4	+0.6	-	-	-	µg/g	(Patel *et al.*, 2018)
Algal extracts (*Kappaphycus alvarezii*)	Open air pot	Salt	Wheat	n	+0.6	+0.7	-	-	-		

Protein hydrolysates (animal)	Greenhouse pot	Drought	Tomato	+0.15	-2.0	+0.35	+0.08	-0.35	+0.9	µg/g	(Casadesús *et al.*, 2019)
Trichoderma harzianum	Seedling nursery	None	Melon shoots	+0.07	+1.3	+0.007	-	+0.8	+0.36	µg/g	(Martínez-Medina *et al.*, 2011)
Glomus mosseae	Seedling nursery	None	Melon shoots	-0.1	n	+0.005	-	n	n		
Trihoderma and *Glomus mosseae*	Seedling nursery	None	Melon shoots	N	n	+0.01	-	n	+0.18		
Glomus intraradices	Greenhouse pot	Drought	Tomato	-	+0.05	-	-	-	-	nmol/g	(Ruiz-Lozano *et al.*, 2016)
Glomus intraradices	Greenhouse pot	Drought	Lettuce	-	+0.04	-	-	-	-		
Azotobacter vinelandii	Pot	Salinity	Rice leaves	+1.2	-	+1.1	+0.9	-	-	µg/g	(Sahoo *et al.*, 2014)
			Rice roots	+1.4	-	+0.2	+1.0	-	-		
Azotobacter vinelandii	Hydroponic system	Heavy metals	Rice roots	+1.3	-	+0.9	+1.8	-	-	µg/g	(Sahoo *et al.*, 2021)
Bacillus licheniformis	Greenhouse pot	None	Onion leaves	-	-	+7.7 (d)	-	-	-	pmol/g	(Gupta *et al.*, 2021)
Pseudomonas fluorenscence	Greenhouse pot	None	Onion leaves	-	-	+10.7 (d)	-	-	-		

Bacillus subtilis	Greenhouse pot	None	Cauliflower	-0.17	n	-	+29	-	+6.0	µg/g	(Ekinci *et al.*, 2014)
Azospirillum brasilense with N	Field	None	Soybean	+0.07	-80000	+0.06	+0.1	-	-	nmol/g	(Zahedi & Abbasi, 2015)

[a]None refers to no applied exogenous compound.

[b]None refers to no stress treatment exposure.

[c]The (-) symbol indicates not reported results, while (n) stands for no detected change(s).

Reference

Ali O, Ramsubhag A, Daniram Benn Jr. Ramnarine S, Jayaraman J. 2022. Transcriptomic changes induced by applications of a commercial extract of Ascophyllum nodosum on tomato plants. *Scientific Reports* 12.

Cabo S, Morais MC, Aires A, Carvalho R, Pascual-Seva N, Silva AP, Gonçalves B. 2020. Kaolin and seaweed-based extracts can be used as middle and long-term strategy to mitigate negative effects of climate change in physiological performance of hazelnut tree. *Journal of Agronomy and Crop Science* 206: 28–42.

Casadesús A, Polo J, Munné-Bosch S. 2019. Hormonal effects of an enzymatically hydrolyzed animal protein-based biostimulant (pepton) in water-stressed tomato plants. *Frontiers in Plant Science* 10.

Chen Y, Jin M, Wu CY, Bao JP. 2022a. Effects of Plant Growth Regulators on the Endogenous Hormone Content of Calyx Development in 'Korla' Fragrant Pear. *HortScience* 57: 497–503.

Chen Q, Qu Z, Ma G, Wang W, Dai J, Zhang M, Wei Z, Liu Z. 2022b. Humic acid modulates growth, photosynthesis, hormone and osmolytes system of maize under drought conditions. *Agricultural Water Management* 263.

Chen J, Zhang NN, Pan Q, Lin XY, Shangguan Z, Zhang JH, Wei GH. 2020. Hydrogen sulphide alleviates iron deficiency by promoting iron availability and plant hormone levels in Glycine max seedlings. *BMC Plant Biology* 20.

Dinler BS, Gunduzer E, Tekinay T. 2016. Pre-treatment of fulvic acid plays a stimulant role in protection of soybean (Glycine max L.) leaves against heat and salt stress. *Acta Biologica Cracoviensia Series Botanica* 58: 29–41.

Du H, Liu H, Xiong L. 2013. Endogenous auxin and jasmonic acid levels are differentially modulated by abiotic stresses in rice. *Frontiers in Plant Science* 4.

Ekinci M, Turan M, Yildirim E. 2014. *Effect of plant growth promoting rhizobacteria on growth, nutrient, organic acid, amino acid and hormone content of cauliflower (Brassica oleracea L. var. botrytis) transplants*.

El-Hoseiny HM, Helaly MN, Elsheery NI, Alam-Eldein SM. 2020. Humic acid and boron to minimize the incidence of alternate bearing and improve the productivity and fruit quality of mango trees. *HortScience* 55: 1026–1037.

Gao F, Li Z, Du Y, Duan J, Zhang T, Wei Z, Guo L, Gong W, Liu Z, Zhang M. 2022. The Combined Application of Urea and Fulvic Acid Solution Improved Maize Carbon and Nitrogen Metabolism. *Agronomy* 12.

Gupta S, Stirk WA, Plačková L, Kulkarni MG, Doležal K, Van Staden J. 2021. Interactive effects of plant growth-promoting rhizobacteria and a seaweed extract on the growth and physiology of Allium cepa L. (onion). *Journal of Plant Physiology* 262.

Hamayun M, Khan SA, Khan AL, Shin JH, Ahmad B, Shin DH, Lee IJ. 2010. Exogenous gibberellic acid reprograms soybean to higher growth and salt stress tolerance. *Journal of Agricultural and Food Chemistry* 58: 7226–7232.

Hosseini SA, Maillard A, Hajirezaei MR, Ali N, Schwarzenberg A, Jamois F, Yvin JC. 2017. Induction of barley silicon transporter HvLsi1 and HvLsi2, increased silicon concentration in the shoot and regulated starch and ABA homeostasis under osmotic stress and concomitant potassium deficiency. *Frontiers in Plant Science* 8.

Khedia J, Dangariya M, Nakum AK, Agarwal P, Panda A, Kumar Parida A, Gangapur DR, Meena R, Agarwal PK. 2020. Sargassum seaweed extract enhances Macrophomina phaseolina resistance in tomato by regulating phytohormones and antioxidative activity.

Latif HH. 2014. *PHYSIOLOGICAL RESPONSES OF PISUM SATIVUM PLANT TO EXOGENOUS ABA APPLICATION UNDER DROUGHT CONDITIONS*.

Lee SK, Sohn EY, Hamayun M, Yoon JY, Lee IJ. 2010. Effect of silicon on growth and salinity stress of soybean plant grown under hydroponic system. *Agroforestry Systems* 80: 333–340.

Martínez-Medina A, Roldán A, Albacete A, Pascual JA. 2011. The interaction with arbuscular mycorrhizal fungi or Trichoderma harzianum alters the shoot hormonal profile in melon plants. *Phytochemistry* 72: 223–229.

Nadeem F, Azhar M, Anwar-ul-Haq M, Sabir M, Samreen T, Tufail A, Awan HUM, Juan W. 2020. Comparative Response of Two Rice (Oryza sativa L.) Cultivars to Applied Zinc and Manganese for Mitigation of Salt Stress. *Journal of Soil Science and Plant Nutrition* 20: 2059–2072.

Nadira UA, Ahmed IM, Wu F, Zhang G. 2016. The regulation of root growth in response to phosphorus deficiency mediated by phytohormones in a Tibetan wild barley accession. *Acta Physiologiae Plantarum* 38.

Patel K, Agarwal P, Agarwal PK. 2018. Kappaphycus alvarezii sap mitigates abiotic-induced stress in Triticum durum by modulating metabolic coordination and improves growth and yield. *Journal of Applied Phycology* 30: 2659–2673.

Paulert R, Ascrizzi R, Malatesta S, Berni P, Noseda MD, de Carvalho MM, Marchioni I, Pistelli L, Duarte MER, Mariotti L, *et al.* 2021. Ulva intestinalis extract acts as biostimulant and modulates metabolites and hormone balance in basil (Ocimum basilicum l.) and parsley (petroselinum crispum l.). *Plants* 10.

Reckova S, Tuma J, Dobrev P, Vankova R. 2019. Influence of copper on hormone content and selected morphological, physiological and biochemical parameters of hydroponically grown Zea mays plants. *Plant Growth Regulation* 89: 191–201.

Réthoré E, Ali N, Yvin JC, Hosseini SA. 2020. Silicon regulates source to sink metabolic homeostasis and promotes growth of rice plants under sulfur deficiency. *International Journal of Molecular Sciences* 21.

Ruiz-Lozano JM, Aroca R, Zamarreño ÁM, Molina S, Andreo-Jiménez B, Porcel R, García-Mina JM, Ruyter-Spira C, López-Ráez JA. 2016. Arbuscular mycorrhizal symbiosis induces strigolactone biosynthesis under drought and improves drought tolerance in lettuce and tomato. *Plant Cell and Environment* 39: 441–452.

Sahoo RK, Ansari MW, Pradhan M, Dangar TK, Mohanty S, Tuteja N. 2014. A novel azotobacter vinellandii (SRIAz3) functions in salinity stress tolerance in rice. *Plant Signaling and Behavior* 9.

Sahoo RK, Rani V, Tuteja N. 2021. Azotobacter vinelandii helps to combat chromium stress in rice by maintaining antioxidant machinery. *3 Biotech* 11.

Shakirova FM, Sakhabutdinova AR, Bezrukova M V, Fatkhutdinova RA, Fatkhutdinova DR. 2003. Changes in the hormonal status of wheat seedlings induced by salicylic acid and salinity.

Sheteiwy MS, Dong Q, An J, Song W, Guan Y, He F, Huang Y, Hu J. 2017. Regulation of ZnO nanoparticles-induced physiological and molecular changes by seed priming with humic acid in Oryza sativa seedlings. *Plant Growth Regulation* 83: 27–41.

Sun X, Hu C, Tan Q, Liu J, Liu H. 2009. Effects of molybdenum on expression of cold-responsive genes in abscisic acid (ABA)-dependent and ABA-independent pathways in winter wheat under low-temperature stress. *Annals of Botany* 104: 345–356.

Torun H, Novák O, Mikulík J, Strnad M, Ayaz FA. 2022. The Effects of Exogenous Salicylic Acid on Endogenous Phytohormone Status in Hordeum vulgare L. under Salt Stress. *Plants* 11.

Wang X, Xu C, Cang J, Zeng Y, Yu J, Liu L, Zhang D, Wang J. 2015. *Effects of Exogenous GA 3 on Wheat Cold Tolerance*.

Wang C, Yang A, Yin H, Zhang J. 2008. Influence of water stress on endogenous hormone contents and cell damage of maize seedlings. *Journal of Integrative Plant Biology* 50: 427–434.

Xing X, Jiang H, Zhou Q, Xing H, Jiang H, Wang S. 2016. Improved drought tolerance by early IAA- and ABA-dependent H2O2 accumulation induced by α-naphthaleneacetic acid in soybean plants. *Plant Growth Regulation* 80: 303–314.

Zahedi H, Abbasi S. 2015. Effect of plant growth promoting rhizobacteria (PGPR) and water stress on phytohormones and polyamines of soybean. *Indian Journal of Agricultural Research* 49: 427–431.

Zhang W, Wang J, Huang Z, Mi L, Xu K, Wu J, Fan Y, Ma S, Jiang D. 2019. Effects of low temperature at booting stage on sucrose metabolism and endogenous hormone contents in winter wheat spikelet. *Frontiers in Plant Science* 10.
